# Mobile *Flowering Locus T* RNA – Biological Relevance and Biotechnological Potential

**DOI:** 10.3389/fpls.2021.792192

**Published:** 2022-01-03

**Authors:** Zhiming Yu, Weiwei Chen, Yue Wang, Pengcheng Zhang, Nongnong Shi, Yiguo Hong

**Affiliations:** ^1^Research Centre for Plant RNA Signaling, College of Life and Environmental Sciences, Hangzhou Normal University, Hangzhou, China; ^2^School of Science and the Environment, University of Worcester, Worcester, United Kingdom; ^3^School of Life Sciences, University of Warwick, Coventry, United Kingdom

**Keywords:** florigen, *Flowering Locus T*, mRNA, long-distancing movement, mobile RNA-based genome editing

## Abstract

Many systemically mobile mRNAs have been revealed in phloem. However, very few of them have been found to be of clear signaling functions. One of such rare examples is the mobile *Flowering locus T* (*FT*) mRNA despite the continuous debate about its mobility and biological relevance to the control of flowering time in plants. Nevertheless, accumulating evidence supports the notion of the long-distance movement of *FT* mRNA from leaf to shoot apex meristem and its role in flowering. In this review, we discuss the discovery of florigenic *FT*, the initial debate on long-distance movement of *FT* mRNA, emerging evidence to prove its mobility, and the use of mobile *FT* mRNA to generate heritable transgenerational gene editing in plants. We elaborate on evidence from virus-based RNA mobility assay, plant grafting, RNA with fluorescent protein labeling, and CRISPR/Cas9 gene-editing technology, to demonstrate that the *FT* mRNA besides the FT protein can move systemically and function as an integral component of the florigenic signal in flowering. We also propose a model to prompt further research on the molecular mechanism underlying the long-distance movement of this important mobile signaling RNA in plants.

## Introduction

Wheat, rice, and maize are the three most important crops which produce seeds as food to feed people globally (Borlaug, 2002). To produce seeds, flowering is a necessary and significant transition from vegetative to reproductive growth in these crop plants (Srikanth and Schmid, 2011). Therefore, flowering is essential not only for plant propagation but also for the survival of humanity. On the other hand, unlike animals, plants rooting in soil cannot move away from surrounding environments and hazards such as biotic or abiotic stresses. To survive and thrive, plants can generate a wide range of responsive signals. Indeed, stress stimulation sensed, and signals perceived by any part of plants can be collected and transported to cells and/or the entire plant through the vascular system (Takahashi and Shinozaki, 2019). The flowering plant vascular system consists of phloem and xylem, where phloem moves materials in a source-to-sink direction, and xylem typically moves materials upward from the roots, to facilitate transportation of these stimulus signals in plants (Deeken et al., 2008; Lucas et al., 2013). Recently, more and more systemically mobile mRNAs have been revealed in phloem. However, up to now, only a few mRNAs with the long-distance movement have been demonstrated to be involved in signal transduction in plant physiological processes (Jackson and Hong, 2012).

To flower, plants perceive the day-length changes in leaves and synthesize a flowering messenger. This signal molecule, dubbed florigen, a theoretical flowering initiation switch, moves long-distance from leave to shoot apical meristem (SAM) through the phloem vascular system to induce flowering (Chailakhyan, 1968; Yanovsky and Kay, 2003; Andrés and Coupland, 2012). However, it took decades to define the nature of florigen, the flowering signaling molecule (Imaizumi and Kay, 2006). In this article, we have discussed the discovery of florigenic *Flowering Locus T* (*FT*), the initial debate on long-distance movement of *FT* mRNA and its biological relevance to flowering, emerging evidence to prove *FT* mRNA mobility, and the application of mobile *FT* mRNA to generate heritable transgenerational gene editing. We also discuss ideas to prompt further investigation into the molecular mechanisms underlying the long-distance movement of this important mobile signaling RNA in plants.

## Role of Florigenic *Flowering Locus T* in the Induction of Flowering

*Flowering Locus T* encodes mobile florigen to induce plant flowering (Evans, 1971; Turck et al., 2008). This is well documented in literature. For instance, an activation tagged T-DNA mutant overexpressed *FT* and flowered early independently of day-length (Kardailsky et al., 1999). Ethyl methane sulfonate-induced point mutations in *FT* such as a single amino acid substitution in *ft-3* (Arg_119_His; Kardailsky et al., 1999) or premature termination in *ft-7* (Trp_138_STOP; Corbesier et al., 2007), led to late flowering. A knockout *FT* mutant *ft-10* in which a T-DNA was inserted in the first intron was also late flowering (Yoo et al., 2005). Moreover, different *Arabidopsis thaliana* ecotypes flowered at a various time dependent on their environmental adaptability. Through quantitative trait locus (QTL) mapping of the recombinant inbred line populations, Schwartz et al. (2009) found that the QTL interval to a 6.7 kb region upstream of the *FT* coding sequence. Tissue-specific expression assays by dissecting the *FT* promoter activities also reveal that *FT* transcription is under the control of CONSTANS, and this occurs only in leaf veins but not the shoot meristem (Adrian et al., 2010). The *FT* transcript level increases under long-day (LD) but decreases under the short-day (SD), consistent with that Arabidopsis plants flowered much earlier in LD than SD growth conditions (Corbesier et al., 2007). It is believed that florigenic *FT* once expressed in leaves travels long distances to SAM to induce flowering. This is consistent with the compelling evidence for the requirement of the movement of FT in the induction of flowering (Abe et al., 2005; Wigge et al., 2005; Jaeger and Wigge, 2007; Mathieu et al., 2007). Taken together, these genetic and molecular analyses have demonstrated the role of FT and the requirement of movement of florigen in floral induction in plants.

## The Debate on the Nature of Mobile Florigen: *Flowering Locus T* mRNA Versus FT Protein

Transcription of *FT* produces mRNA that then translates into the FT protein in leaves. It is no doubt that the FT protein is essential for cell-autonomous function in induction of flowering when it presents in SAM. However, the burning question is whether the FT protein or the *FT* mRNA is non-cell autonomous and directly contributes to the mobile florigenic signal. It is worthwhile noting that both protein and RNA (even DNA) can spread from cell to cell and over long-distance in plants. For instance, plant RNA and DNA viruses are long known to move their RNA and DNA genomes intercellular and systemically. Moreover, viruses express movement proteins that are required to promote the intercellular and systemic spread of viral RNA or DNA in plants (Qin et al., 2015). Thus, it is reasonable to presume that *FT* mRNA, its protein product, or both can contribute to florigen. Nevertheless, the initial finding that the *FT* mRNA produced under a heat shock-inducible promoter in distal leaf tissues can trigger flowering in SAM, attributing the *FT* mRNA as the non-cell autonomous mobile florigenic signal (Huang et al., 2005; Hurtley and Szuromi, 2005). However, this work cannot exclude the possibility of the potential role of the FT protein in the non-cell autonomous mobile florigenic signaling. Even more unfortunately, key data to support *FT* mRNA mobility were brought into question and this work has been retracted although heat treatment of local leaf was sufficient to induce flowering (Böhlenius et al., 2007).

In the meanwhile, the FT protein was reported to move long-distance and induce flowering in plants. For example, in LD *Arabidopsis* the FT-GFP fusion protein was shown to move across the grafting junction (Corbesier et al., 2007). On the other hand, in SD rice, Hd3a, the rice ortholog of *Arabidopsis FT*, is expressed in blade tissue, but interacts with SAM-specific FD, heralding that the FT protein spreads to SAM through vascular tissue (Tamaki et al., 2007). Recently, FT protein has been found to move from companion cells to sieve elements (Liu et al., 2020). However, whether FD is strictly SAM-specific is questionable since FD expression can also be detected in mature leaf tissues (Klepikova et al., 2016). This experiment suggests that it remains possible for both *FT* and *FD* RNAs to move to and then be translated into proteins in SAM (Pennisi, 2007). Thus, the nature of mobile florigen, *FT* mRNA vs FT protein, remains debatable.

## The Evidence on Mobile *Flowering Locus T* mRNA: To Move or Not to Move

### Virus-Based RNA Mobility Assay

As aforementioned, plant viruses can move their RNA and DNA genomes from cell to cell and over a long distance. This is determined (at least in part) by virus-encoded movement proteins. Thus, defects in viral movement proteins can rid viruses of intercellular and systemic mobility whilst such movement-deficient viruses can still replicate in single infected cells. Based on these, two plant RNA viruses, i.e., *Potato virus X* (PVX, Chapman et al., 1992) and *Turnip crinkle virus* (TCV; Ryabov et al., 2004), were modified as RNA mobility Assay (RMA) vectors in which the coat protein gene was deleted from each virus genome. The resultant virus-based RMA vectors PVX/ΔCP and TCV/ΔCP, can infect, but are restricted within individual leaf epidermal cells (Li et al., 2009). By engineering the *FT* RNA into the two RMA vectors, it restores cell-to-cell and long-distance movement of PVX/ΔCP and TCV/ΔCP RNA. Such virus-based RMAs provide compelling answers to three questions on *FT* mRNA movement. Firstly, *FT* mRNA can move long distances independent of FT protein. This conclusion was also confirmed by virus-free RMA in which *FT* RNA produced *via* agro-infiltration of local leaf tissues can spread to distal non-infiltrated newly growing leaves (Li et al., 2009). Secondly, the core mobile determinant consists of 102-nucleotides at the 5′ end of the *FT mRNA* (Li et al., 2009). Thirdly, *FT mRNA* can facilitate PVX entry of SAM where viruses are usually excluded (Wu et al., 2020), and leads to virus-induced gene silencing in SAM. Moreover, the long-distance *FT RNA* movement is also shown to enhance early flowering (Li et al., 2011). It is worthwhile noting that the virus-based RMA is also used to show that long-distance movement of the *BEL5* mRNA contributes to tuberization in potato (Cho et al., 2016).

### Grafting Evidence

Grafting is one of the gold standard methods to study long-distance RNA movement (Gaut et al., 2019). Using heterografting technique coupled with RNAseq, 2,006 genes producing mobile mRNAs were identified in two different *A. thaliana* ecotypes (Thieme et al., 2015), and 138 *Arabidopsis* mobile mRNAs were found in *A. thaliana* (as stock) - *Nicotiana benthamiana* (as scion) system (Notaguchi et al., 2015). However, it is conceivable that this method is more suitable to identify mobile RNAs of high abundance. Therefore, it is unsurprising that *FT* mRNA with limited and dynamic expression pattern cannot be easily detected in previous studies (Corbesier et al., 2007; Tamaki et al., 2007). To avoid this issue, some mature leaves of the *ft-3* scion grafted onto the wild-type stock were removed in order to enrich potential wild-type *FT* mRNAs originated from wild-type stock. This indeed allows a positive detection of the wild-type *FT* mRNA movement from stock to scion through grafting junction (Lu et al., 2012). Furthermore, such grafting experiment also led to identify the 210-nt sequence at the 5′ end of *FT* mRNA as the mobility determinant (Lu et al., 2012), consistent with the virus-based RMA’s findings (Li et al., 2009).

### Intracellular RNA Imaging

The bacteriophage coat protein MS2 can bind its target RNAs “stem-loop repeats (SL).” The MS2-GFP fusion protein is often used to label and image RNAs in different organisms (Querido and Chartrand, 2008). However, due to its high background fluorescent noise in the cytoplasm, this method is not extensively used in plants. Nevertheless, MS2 was found to be very specific nucleus localized when it is fused with transcription factor FD (Luo et al., 2018). The nuclear retention of MS2_FD_-GFP was much longer than MS2_SV40_-GFP. Luo et al. (2018) then co-expressed the MS2_FD_-GFP, and chimeric *SL-FT* mRNA. Through fluorescent imaging, the *SL-FT* mRNA was found to move intracellularly and mainly accumulated at the plasmodesmata sites; however, the dynamic process of the *SL-FT* mRNA moving from one cell to another was not observed (Luo et al., 2018).

### Mobile *Flowering Locus T* mRNA-Assisted Seed Transmission of Gene Editing – Biotechnological Potential

Clustered regularly interspaced short palindromic repeats (CRISPR)/CRISPR-associated protein 9 (CRISRP/Cas9) gene editing system has revolutionized targeted gene editing (Jinek et al., 2012; Cong et al., 2013; Feng et al., 2013). This gene editing tool contains three main components, Cas9 enzyme, spacer sequence and sgRNA (Jinek et al., 2012). Spacer and sgRNA are usually taken as a whole. As for plants, agrobacterium-mediated CRISPR/Cas9 technology is widely used. However, this technology is often involved in genetically modified plants and time-consuming screen of homologous lines with the edited target gene. These issues can be partially avoided by a technology so called virus-induced gene/genome editing (ViGE; Ali et al., 2015).

However, gene editing resulted from ViGE is often not heritable to next-generation due to virus exclusion from SAM, thus its use is limited (Zhang et al., 2020). How to generate heritable ViGE is the issue that needs to be resolved. Recently, Ellison et al. (2020) have elegantly exploited the fact that the mobile translatable and non-translatable *FT* RNA can facilitate RNA virus entering SAM (Li et al., 2011), and used the *Tobacco rattle virus* (TRV) to deliver sgRNA-tagged with the mobile *FT* RNA into SAM. In the way, the mutant progeny of *Nicotiana benthamiana* were recovered in next-generation at frequencies ranging from 65 to 100%; and due to sgRNA-targeting of *PDS* gene, the transgenerational seedlings with albino phenotype resulted from the *PDS* gene editing *via* TRV/(m)*FT-sgRNA* were statistically different compared with the mock control (Ellison et al., 2020). This demonstrated that the mobile *FT* RNA can promote the *PDS* sgRNA into SAM and enhances the progeny gene editing efficiency of the CRISPR/Cas9 system (Ellison et al., 2020). Similar results have been also reported using PVX to deliver *FT* RNA-tagged sgRNA to plants (Uranga et al., 2021).

Moreover, a cotton leaf crumple virus (CLCrV)-mediated ViGE system was also developed. In this case, sgRNAs were fused to the 102-nt *FT* mRNA, then expressed by CLCrV in transgenic *Cas9 A. thaliana*. The enhanced gene editing efficiency of 4.35–8.79% was found in progeny plants free of the CLCrV genome (Lei et al., 2021). Together, all these latest evidence shows that *FT* RNA is mobile and can enter SAM, as well as that such mobile RNA element has a high potential of biotechnological application in inheritable and transgenerational genome editing in plants and crops.

### Movement of *Flowering Locus T* Homolog Genes

*Flowering Locus T* is one of six phosphatidyl ethanolamine-binding protein (PEBP) family members and the other five are TERMINAL FLOWER1 (TFL1) LIKE, MOTHER OF FT AND TFL1 (MFT), BROTHER OF FT AND TFL1 (BFT), ARABIDOPSIS THALIANA CENTRORADIALIS (ATC), and TWIN SISTER OF FT (TSF) in Arabidopsis (Karlgren et al., 2011). Phylogenetic analysis indicates that this small multigene family consists of three classes, FT-LIKE (FT and TSF), MFT, and TFL1-LIKE (ATC, BFT, and TFL1) (Chardon and Damerval, 2005). FT and TFL1 are two highly conserved homologous proteins, which have opposite functions but compete to regulate the initiation of plant flowering (Hanzawa et al., 2005). So far, there is no evidence that *TFL1* RNA can move long distance. On the other hand, *CET1* mRNA, an ortholog of the Arabidopsis antiflorigen *ATC*, is mobile, as revealed in tobacco/Arabidopsis grafting experiments. Its non-cell-autonomously movement is also confirmed in heterograft of tobacco and tomato (Huang et al., 2018). Other *FT* ortholog gene mRNAs were also found moving across the tomato-tobacco heterograft junction (Huang et al., 2018).

## What Determines the Mobility of *Flowering Locus T* mRNA?

### Primary *Flowering Locus T* RNA Structures

Synonymous codon substitution in *FT* (*synFT*) does not change the FT protein amino acid sequence but alters the RNA sequence. Notaguchi et al. (2008) transformed *ft-1* with an expression cassette in which *synFT* contains 171 (of 175) codon substitutions. In grafting experiments where *ft-1* was used as a recipient stock and transgenic *synFT/ft-1* as a donor scion, the *synFT mRNA* was not detectable in the *ft-1* shoot apical region. These experiments also suggest that changes in the *FT* RNA sequence did not affect the FT protein movement (Notaguchi et al., 2008). However, unlike the later grafting study (Lu et al., 2012), no enrichment of potential mobile *synFT* mRNA was done. This may explain why the *synFT* RNA could not be detected (Notaguchi et al., 2008). On the other hand, changes of primary mRNA sequence may lead to alternation of secondary structures (see below), which may be required for systemic *FT* RNA movement.

### RNA Secondary Structures

A high number of tRNAs were detected in the phloem sap of pumpkin (*Cucurbita maxima*). Among these mobile tRNAs, their distributions are uneven. For example, no Ile-tRNA or very few Arg-tRNA but a few Asp-tRNA molecules were detected; however, Cys, Leu, Phe, Try, Trp, and Ser tRNAs were predominantly present in the phloem sap (Zhang et al., 2009). These tRNAs have been predicted to serve as long-distance signals (Zhang et al., 2009, 2016; Lezzhov et al., 2019; Wang et al., 2021), suggesting that tRNA or tRNA-like structure could be an important factor, if not the determinant for systemic RNA movement. Indeed, in the ViGE systems, like the *FT* mRNA/102 nt-*FT* RNA element, Met, Gly, and Ile tRNAs were also found to improve transgenerational gene editing when they were fused to sgRNA (Ellison et al., 2020). It is possible that the *FT* mRNA may form unique structure, or tRNA/tRNA-like secondary or even tertiary structures that are important for its mobility.

### RNA Binding Proteins

In plants, it is well-established that the movement of RNA requires RNA binding proteins. For example, KNOTTED1 (KN1) (Lucas et al., 1995) and a viral movement paralog protein in *Cucurbita maxima*, CmPP16 have been reported to be involved in cellular RNA trafficking (Xoconostle-Cázares et al., 1999). In *Arabidopsis*, a conserved SMALL RNA-BINDING PROTEIN 1 (SRBP1) family member, AtSRBP1, was found to mediate small RNA movement. AtSRBP1, a glycine-rich (GR) RNA-binding protein, also named AtGRP7, can bind to single-stranded siRNA (Yan et al., 2020). It is plausible that *FT* mRNA movement may also involve certain RNA binding protein(s) (Jackson and Hong, 2012).

### RNA Epigenetic Modification

Numerous RNA modifications have been reported (Boccaletto et al., 2018) and some specific modifications are closely associated with biological functions (Chmielowska-Bąk et al., 2019). In plants, mRNA N6-methyladenosine (m^6^A) and 5-methylcytosine (m^5^C) play crucial and dynamic roles in embryo development, leaf morphogenesis, and root development (Liang et al., 2020). Recently, epigenetic modifications such as m^5^C have been found to be linked with RNA mobility and m^5^C RNAs are predominant in the phloem (Yang et al., 2019; Wang et al., 2021). Interestingly, in RNA m^5^C methylation-deficient mutants, mobile *TRANSLATIONALLY CONTROLLED TUMOR PROTEIN 1* and *HEAT SHOCK COGNATE PROTEIN 70.1* mRNAs become immobile (Yang et al., 2019). We speculate that m^5^C or other types of epigenetic modifications may play a role in *FT mRNA* mobility.

## Prospective

**FIGURE 1:**
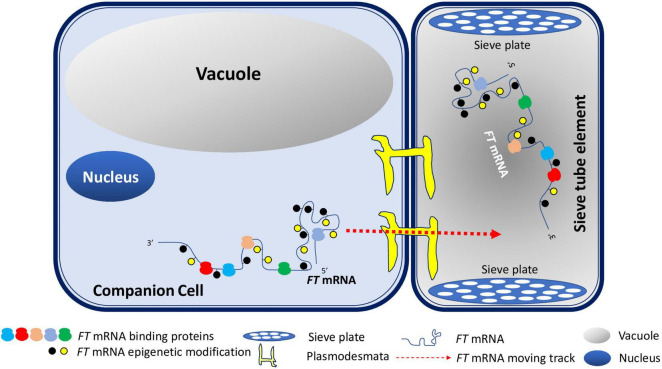
A Model for *FT* mRNA Signaling. In companion cells, *FT* mRNAs may be epigenetically modified and form specific structures such as tRNA-like secondary structures. Such structured RNAs may then be recognized by RNA-binding proteins and transported through plasmodesmata into sieve tube. *FT* mRNAs will travel pass sieve plates through phloem to germline cells in shoot apical meristem to induce flowering along with other floral induction factors.

Specificized xylem and phloem are two conduits of the plant transportation system. They not only provide physical support for plants, but also are crucial to transport nutrients, minerals, and various signaling molecules in plants (De Rybel et al., 2016). Phloem consists of two types of cells: companion cells and sieve tubes. Sieve tubes lack nuclei. Almost all inorganic and organic substances are transferred from companion cells to sieve tubes *via* plasmodesmata (Slewinski et al., 2013). While local FT protein produced in source leaf tissues moves into SAM *via* the phloem-transportation highway, emerging evidence demonstrates that *FT* RNA can also undergo the same voyage *en route* to SAM. Therefore, it is possible that both *FT* mRNA and FT protein contribute to the florigen signal. Based on recent findings on the relevance of epigenetic RNA modifications, primary ribonucleotide sequences, secondary structures, and RNA binding proteins to RNA mobility, we propose a model for *FT* mRNA signaling in flowering (Figure 1). Once *FT* mRNAs is produced in companion cells, *FT* mRNAs may be epigenetically modified and form specific structures such as tRNA-like structures. Structured *FT* RNAs may then be recognized by RNA-binding proteins and transported through plasmodesmata into sieve tube. Along with the FT protein, *FT* mRNAs will move across sieve plates through phloem to germline cells in shoot apical meristem to induce flowering together with other floral induction factors. This model explains the possible movement of *FT* mRNA from specific companion cells to sieve tubes, then to distal SAM where they coordinate with other flowering induction factors to induce flowering (Figure 1). This model is also expected to be universal for the study of the mechanism of mobile mRNA in plants.

## Author Contributions

ZY and WC conceived the idea and wrote the draft of the manuscript. YH revised and finalized the manuscript. YW, PZ, and NS were involved in the discussion and helped to wrote the manuscript. All authors contributed to the article and approved the submitted version.

## Conflict of Interest

The authors declare that the research was conducted in the absence of any commercial or financial relationships that could be construed as a potential conflict of interest.

## Publisher’s Note

All claims expressed in this article are solely those of the authors and do not necessarily represent those of their affiliated organizations, or those of the publisher, the editors and the reviewers. Any product that may be evaluated in this article, or claim that may be made by its manufacturer, is not guaranteed or endorsed by the publisher.

## References

[B1] Abe M. Kobayashi Y. Yamamoto S. Daimon Y. Yamaguchi A. Ikeda Y. (2005). FD, a bZIP protein mediating signals from the floral pathway integrator FT at the shoot apex. *Science* 12 1052–1056. 10.1126/science.1115983 16099979

[B2] Adrian J. Farrona S. Reimer J. J. Albani M. C. Coupland G. Turck F. (2010). Cis-Regulatory elements and chromatin state coordinately control temporal and spatial expression of Flowering Locus T in *Arabidopsis*. *Plant Cell* 22 1425–1440. 10.1105/tpc.110.074682 20472817 PMC2899882

[B3] Ali Z. Abul-faraj A. Li L. X. Ghosh N. Piatek M. Mahjoub A. (2015). Efficient virus-mediated genome editing in plants using the CRISPR/Cas9 system. *Mol. Plant* 8 1288–1291. 10.1016/j.molp.2015.02.011 25749112

[B4] Andrés F. Coupland G. (2012). The genetic basis of flowering responses to seasonal cues. *Nat. Rev. Genet.* 13 627–639. 10.1038/nrg3291 22898651

[B5] Boccaletto P. Machnicka M. A. Purta E. Piątkowski P. Bagiński B. Wirecki T. K. (2018). MODOMICS: a database of RNA modification pathways. *Nucleic Acids Res.* 46 D303–D307. 10.1093/nar/gkx1030 29106616 PMC5753262

[B6] Böhlenius H. Eriksson S. Parcy F. Nilsson O. (2007). Retraction. *Science* 316:367. 10.1126/science.316.5823.367b 17446370

[B7] Borlaug N. E. (2002). Feeding a World of 10 Billion people: the miracle ahead. *In Vitro* Cell. *Dev. Biol. Plant* 38 221–228.

[B8] Chailakhyan M. K. (1968). Internal factors of plant flowering. *Annu. Rev. Plant Physiol.* 19 1–37. 10.1146/annurev.pp.19.060168.000245

[B9] Chapman S. Kavanagh T. Baulcombe D. (1992). Potato virus X as a vector for gene expression in plants. *Plant J.* 2 549–557. 10.1046/j.1365-313x.1992.t01-24-00999.x 1344890

[B10] Chardon F. Damerval C. (2005). Phylogenomic analysis of the PEBP gene family in cereals. *J. Mol. Evol.* 61 579–590. 10.1007/s00239-004-0179-4 16170456

[B11] Chmielowska-Bąk J. Arasimowicz-Jelonek M. Deckert J. (2019). In search of the mRNA modification landscape in plants. *BMC Plant Biol.* 19:421. 10.1186/s12870-019-2033-2 31610789 PMC6791028

[B12] Cho S. K. Sharma P. Butler N. M. Kang I. H. Shah S. Rao A. G. (2016). Polypyrimidine tract-binding proteins of potato mediate tuberization through an interaction with StBEL5 RNA. *J. Exp. Bot.* 66 6835–6847. 10.1093/jxb/erv389 26283046 PMC4623692

[B13] Cong L. Ran F. A. Cox D. Lin S. Barretto R. Habib N. (2013). Multiplex genome engineering using CRISPR/Cas systems. *Science* 339 819–823. 10.1126/science.1231143 23287718 PMC3795411

[B14] Corbesier L. Vincent C. Jang S. Fornara F. Fan Q. Searle I. (2007). FT protein movement contributes to long-distance signaling in floral induction of *Arabidopsis*. *Science* 316 1030–1033. 10.1126/science.1141752 17446353

[B15] De Rybel B. Mähönen A. P. Helariutta Y. Weijers D. (2016). Plant vascular development: from early specification to differentiation. *Nat. Rev. Mol. Cell Biol.* 17 30–40. 10.1038/nrm.2015.6 26580717

[B16] Deeken R. Ache P. Kajahn I. Klinkenberg J. Bringmann G. Hedrich R. (2008). Identification of *Arabidopsis thaliana* phloem RNAs provides a search criterion for phloem-based transcripts hidden in complex datasets of microarray experiments. *Plant J.* 55 746–759. 10.1111/j.1365-313X.2008.03555.x 18485061

[B17] Ellison E. E. Nagalakshmi U. Gamo M. E. Huang P. J. Dinesh-Kumar S. Voytas D. F. (2020). Multiplexed heritable gene editing using RNA viruses and mobile single guide RNAs. *Nat. Plants* 6 620–624. 10.1038/s41477-020-0670-y 32483329

[B18] Evans L. T. (1971). Flower induction and the florigen concept. *Annu. Rev. Plant Physiol.* 22 365–394. 10.1186/1472-6750-11-36 21481273 PMC3080291

[B19] Feng Z. Y. Zhang B. T. Ding W. N. Liu X. D. Yang D. L. Wei P. L. (2013). Efficient genome editing in plants using a CRISPR/Cas system. *Cell Res.* 23 1229–1232. 10.1038/cr.2013.114 23958582 PMC3790235

[B20] Gaut B. S. Miller A. J. Seymour D. K. (2019). Living with two genomes: grafting and its implications for plant genome-to-genome interactions, phenotypic variation, and evolution. *Annu. Rev. Genet.* 53 195–215. 10.1146/annurev-genet-112618-043545 31424971

[B21] Hanzawa Y. Money T. Bradley D. (2005). A single amino acid converts a repressor to an activator of flowering. *Proc. Natl. Acad. Sci. U.S.A.* 102 7748–7753. 10.1073/pnas.0500932102 15894619 PMC1140427

[B22] Huang N. C. Luo K. R. Yu T. S. (2018). Mobility of antiflorigen and PEBP mRNAs in tomato-tobacco heterografts. *Plant Physiol.* 178 783–794. 10.1104/pp.18.00725 30150303 PMC6181055

[B23] Huang T. Böhlenius H. Eriksson S. Parcy F. Nilsson O. (2005). The mRNA of the *Arabidopsis* gene FT moves from leaf to shoot apex and induces flowering. *Science* 309 1694–1696. 10.1126/science.1117768 16099949

[B24] Hurtley S. Szuromi P. (2005). The message is the messenger. *Science* 309:1647. 10.1126/science.2005.309.5741.twis

[B25] Imaizumi T. Kay S. A. (2006). Photoperiodic control of flowering: not only by coincidence. *Trends Plant Sci.* 11 550–558. 10.1016/j.tplants.2006.09.004 17035069

[B26] Jackson S. Hong Y. (2012). Systemic movement of FT mRNA and a possible role in floral induction. *Front. Plant Sci.* 3:127. 10.3389/fpls.2012.00127 22701465 PMC3373133

[B27] Jaeger K. E. Wigge P. A. (2007). FT protein acts as a long-range signal in *Arabidopsis*. *Curr. Biol.* 17 1050–1054. 10.1016/j.cub.2007.05.008 17540569

[B28] Jinek M. Chylinski K. Fonfara I. Hauer M. Doudna J. A. Charpentier E. (2012). A programmable dual-RNA-guided DNA endonuclease in adaptive bacterial immunity. *Science* 337 816–821. 10.1126/science.1225829 22745249 PMC6286148

[B29] Kardailsky I. Shukla V. K. Ahn J. H. Dagenais N. Christensen S. K. Nguyen J. T. (1999). Activation tagging of the floral inducer FT. *Science* 286 1962–1965. 10.1126/science.286.5446.1962 10583961

[B30] Karlgren A. Gyllenstrand N. Källman T. Sundström J. F. Moore D. Lascoux M. (2011). Evolution of the PEBP gene family in plants: functional diversification in seed plant evolution. *Plant Physiol.* 156 1967–1977. 10.1104/pp.111.176206 21642442 PMC3149940

[B31] Klepikova A. V. Kasianov A. S. Gerasimov E. S. Logacheva M. D. Penin A. A. (2016). A high resolution map of the *Arabidopsis thaliana* developmental transcriptome based on RNA-seq profiling. *Plant J.* 88 1058–1070. 10.1111/tpj.13312 27549386

[B32] Lei J. F. Dai P. H. Li Y. Zhang W. Q. Zhou G. T. Liu C. (2021). Heritable gene editing using FT mobile guide RNAs and DNA viruses. *Plant Methods* 17:20. 10.1186/s13007-021-00719-4 33596981 PMC7890912

[B33] Lezzhov A. A. Atabekova A. K. Tolstyko E. A. Lazareva E. A. Solovyev A. G. (2019). RNA phloem transport mediated by pre-miRNA and viral tRNA-like structures. *Plant Sci.* 284 99–107. 10.1016/j.plantsci.2019.04.005 31084885

[B34] Li C. Gu M. Shi N. Zhang H. Yang X. Osman T. (2011). Mobile FT mRNA contributes to the systemic florigen signalling in floral induction. *Sci. Rep.* 1 1–6. 10.1038/srep00073 22355592 PMC3216560

[B35] Li C. Zhang K. Zeng X. Jackson S. Zhou Y. Hong Y. (2009). A cis element within Flowering Locus T mRNA determines its mobility and facilitates trafficking of heterologous viral RNA. *J. Virol.* 83 3540–3548. 10.1128/JVI.02346-08 19193810 PMC2663265

[B36] Liang Z. Riaz A. Chachar S. Ding Y. K. Du H. Gu X. F. (2020). Epigenetic modifications of mRNA and DNA in plants. *Mol. Plant* 13 14–30. 10.1016/j.molp.2019.12.007 31863849

[B37] Liu L. Zhang Y. Yu H. (2020). Florigen trafficking integrates photoperiod and temperature signals in *Arabidopsis*. *J. Integr. Plant Biol.* 62 1385–1398. 10.1111/jipb.13000 32729982

[B38] Lu K. J. Huang N. C. Liu Y. S. Lu C. A. Yu T. S. (2012). Long-distance movement of *Arabidopsis* FLOWERING LOCUS T RNA participates in systemic floral regulation. *RNA Biol.* 9 653–662. 10.4161/rna.19965 22614833

[B39] Lucas W. J. Bouche-Pillon S. Jackson D. P. Nguyen L. Baker L. Ding B. (1995). Selective trafficking of KNOTTED1 homeodomain protein and its mRNA through plasmodesmata. *Science* 270 1980–1983. 10.1126/science.270.5244.1980 8533088

[B40] Lucas W. J. Groover A. Lichtenberger R. Furuta K. Yadav S. R. Helariutta Y. (2013). The plant vascular system: evolution, development and functions. *J. Integr. Plant Biol.* 55 294–388. 10.1111/jipb.12041 23462277

[B41] Luo K. R. Huang N. C. Yu T. S. (2018). Selective targeting of mobile mRNAs to plasmodesmata for cell-to-cell movement. *Plant Physiol.* 177 604–614. 10.1104/pp.18.00107 29581179 PMC6001314

[B42] Mathieu J. Warthmann N. Küttner F. Schmid M. (2007). Export of FT protein from phloem companion cells is sufficient for floral induction in *Arabidopsis*. *Curr. Biol.* 17 1055–1060. 10.1016/j.cub.2007.05.009 17540570

[B43] Notaguchi M. Abe M. Kimura T. Daimon Y. Kobayashi T. Yamaguchi A. (2008). Long-distance, graft-transmissible action of *Arabidopsis* flowering locus T protein to promote flowering. *Plant Cell Physiol.* 49 1645–1658. 10.1093/pcp/pcn154 18849573

[B44] Notaguchi M. Higashiyama T. Suzuki T. (2015). Identification of mRNAs that move over long distances using an RNA-Seq analysis of *Arabidopsis*/Nicotiana benthamiana heterografts. *Plant Cell Physiol.* 56 311–321. 10.1093/pcp/pcu210 25527829

[B45] Pennisi E. (2007). Plant science. Long-sought plant flowering signal unmasked, again. *Science* 316 350–351. 10.1126/science.316.5823.350 17446357

[B46] Qin C. Zhang Q. He M. Kong J. Li B. Mohamed A. (2015). *Applied Plant Genomics and Biotechnology.* Witney: Woodhead Publishing.

[B47] Querido E. Chartrand P. (2008). Using fluorescent proteins to study mRNA trafficking in living cells. *Methods Cell Biol.* 85 273–292. 10.1016/S0091-679X(08)85012-1 18155467

[B48] Ryabov E. V. Van Wezel R. Walsh J. Hong Y. (2004). Cell-to-cell, but not long-distance, spread of RNA silencing that is induced in individual epidermal cells. *J. Virol.* 78 3149–3154. 10.1128/jvi.78.6.3149-3154.2004 14990735 PMC353744

[B49] Schwartz C. Balasubramanian S. Warthmann N. Michael T. P. Lempe J. Sureshkumar S. (2009). Cis-regulatory changes at FLOWERING LOCUS T mediate natural variation in flowering responses of *Arabidopsis thaliana*. *Genetics* 183 723–732. 10.1534/genetics.109.104984 19652183 PMC2766330

[B50] Slewinski T. L. Zhang C. Turgeon R. (2013). Structural and functional heterogeneity in phloem loading and transport. *Front. Plant Sci.* 5:244. 10.3389/fpls.2013.00244 23847646 PMC3701861

[B51] Srikanth A. Schmid M. (2011). Regulation of flowering time: all roads lead to Rome. *Cell. Mol. Life Sci.* 68 2013–2037. 10.1007/s00018-011-0673-y 21611891 PMC11115107

[B52] Takahashi F. Shinozaki K. (2019). Long-distance signaling in plant stress response. *Curr. Opin. Plant Biol.* 47 106–111. 10.1016/j.pbi.2018.10.006 30445314

[B53] Tamaki S. Matsuo S. Wong H. L. Yokoi S. Shimamoto K. (2007). Hd3a protein is a mobile flowering signal in rice. *Science* 316 1033–1036. 10.1126/science.1141753 17446351

[B54] Thieme C. J. Rojas-Triana M. Stecyk E. Schudoma C. Zhang W. Yang L. (2015). Endogenous *Arabidopsis* messenger RNAs transported to distant tissues. *Nat. Plants* 1 1–9. 10.1038/nplants.2015.25 27247031

[B55] Turck F. Fornara F. Coupland G. (2008). Regulation and identity of florigen: FLOWERING LOCUS T moves center stage. *Annu. Rev. Plant Biol.* 59 573–594. 10.1146/annurev.arplant.59.032607.092755 18444908

[B56] Uranga M. Aragonés V. Selma S. Vázquez-Vilar M. Orzáez D. Daròs J. A. (2021). Efficient Cas9 multiplex editing using unspaced sgRNA arrays engineering in a Potato virus X vector. *Plant J.* 106 555–565. 10.1111/tpj.15164 33484202 PMC8251967

[B57] Wang T. Li X. J. Zhang X. J. Wang Q. Liu W. Q. Lu X. H. (2021). RNA motifs and modification involve in RNA long-distance transport in plants. *Front. Cell Dev. Biol.* 9:651278. 10.3389/fcell.2021.651278 33869208 PMC8047152

[B58] Wigge P. A. Kim M. C. Jaeger K. E. Busch W. Schmid M. Lohmann J. U. (2005). Integration of spatial and temporal information during floral induction in *Arabidopsis*. *Science* 309 1056–1059. 10.1126/science.1114358 16099980

[B59] Wu H. Qu X. Dong Z. Luo L. Shao C. Forner J. (2020). WUSCHEL triggers innate antiviral immunity in plant stem cells. *Science* 370 227–231. 10.1126/science.abb7360 33033220

[B60] Xoconostle-Cázares B. Xiang Y. Ruiz-Medrano R. Wang H. L. Monzer J. Yoo B. C. (1999). Plant paralog to viral movement protein that potentiates transport of mRNA into the phloem. *Science* 283 94–98. 10.1126/science.283.5398.94 9872750

[B61] Yan Y. Ham B. K. Chong Y. H. Yeh S. D. Lucas W. J. (2020). A plant small RNA-binding protein 1 family mediates cell-to-cell trafficking of RNAi signals. *Mol Plant* 13 321–335. 10.1016/j.molp.2019.12.001 31812689

[B62] Yang L. Perrera V. Saplaoura E. Apelt F. Bahin M. Kramdi A. (2019). m5C methylation guides systemic transport of messenger RNA over graft junctions in plants. *Curr. Biol.* 29 2465.e5–2476.e5. 10.1016/j.cub.2019.06.042 31327714

[B63] Yanovsky M. J. Kay S. A. (2003). Living by the calendar: how plants know when to flower. *Nat. Rev. Mol. Cell Biol.* 4 265–276. 10.1038/nrm1077 12671649

[B64] Yoo S. K. Chung K. S. Kim J. Lee J. H. Hong S. M. Yoo S. J. (2005). CONSTANS activates SUPPRESSOR OF OVEREXPRESSION OF CONSTANS 1 through FLOWERING LOCUS T to promote flowering in *Arabidopsis*. *Plant Physiol.* 139 770–778. 10.1104/pp.105.066928 16183837 PMC1255994

[B65] Zhang S. D. Sun L. Kragler F. (2009). The phloem-delivered RNA pool contains small noncoding RNAs and interferes with translation. *Plant Physiol.* 150 378–387. 10.1104/pp.108.134767 19261735 PMC2675743

[B66] Zhang W. N. Thieme C. J. Kollwig G. Apelt F. Yang L. Winter N. (2016). tRNA-related sequences trigger systemic mRNA transport in plants. *Plant Cell* 28 1237–1249. 10.1105/tpc.15.01056 27268430 PMC4944404

[B67] Zhang X. Kang L. H. Zhang Q. Meng Q. Q. Pan Y. F. Yu Z. M. (2020). An RNAi suppressor activates in planta virus-mediated gene editing. *Funct. Integr. Genomics* 20 471–477. 10.1007/s10142-019-00730-y 31848794

